# Photogrammetry Applied to Neurosurgery: A Literature Review

**DOI:** 10.7759/cureus.46251

**Published:** 2023-09-30

**Authors:** Martin Trandzhiev, Donika I Vezirska, Ivan Maslarski, Milko D Milev, Lili Laleva, Vladimir Nakov, Jan F Cornelius, Toma Spiriev

**Affiliations:** 1 Department of Neurosurgery, Acibadem City Clinic University Hospital Tokuda, Sofia, BGR; 2 Department of Anatomy and Histology, Pathology, and Forensic Medicine, University Hospital Lozenetz, Medical Faculty, Sofia University, Sofia, BGR; 3 Department of Neurosurgery, University Hospital of Düsseldorf, Heinrich Heine University, Düsseldorf, DEU

**Keywords:** scoliosis, craniosynostosis, neurosurgical approaches, three-dimensional models, neurosurgery, photogrammetry

## Abstract

Photogrammetry refers to the process of creating 3D models and taking measurements through the use of photographs. Photogrammetry has many applications in neurosurgery, such as creating 3D anatomical models and diagnosing and evaluating head shape and posture deformities. This review aims to summarize the uses of the technique in the neurosurgical practice and showcase the systems and software required for its implementation. A literature review was done in the online database PubMed. Papers were searched using the keywords “photogrammetry”, “neurosurgery”, “neuroanatomy”, “craniosynostosis” and “scoliosis”. The identified articles were later put through primary (abstracts and titles) and secondary (full text) screening for eligibility for inclusion. In total, 86 articles were included in the review from 315 papers identified. The review showed that the main uses of photogrammetry in the field of neurosurgery are related to the creation of 3D models of complex neuroanatomical structures and surgical approaches, accompanied by the uses for diagnosis and evaluation of patients with structural deformities of the head and trunk, such as craniosynostosis and scoliosis. Additionally, three instances of photogrammetry applied for more specific aims, namely, cervical spine surgery, skull-base surgery, and radiosurgery, were identified. Information was extracted on the software and systems used to execute the method. With the development of the photogrammetric method, it has become possible to create accurate 3D models of physical objects and analyze images with dedicated software. In the neurosurgical setting, this has translated into the creation of anatomical teaching models and surgical 3D models as well as the evaluation of head and spine deformities. Through those applications, the method has the potential to facilitate the education of residents and medical students and the diagnosis of patient pathologies.

## Introduction and background

The word “photogrammetry” describes the process of creating three-dimensional (3D) models from standard two-dimensional (2D) photography with measurable quantitative data [1,2].

In the neurosurgical field, the applications of photogrammetry correlate with the technique’s main functions - 3D model creation, mainly used for the visualization of neurosurgical anatomy [1-22] and for skeletal and bony deformity evaluation [23-83].

The process normally involves a series of steps, with the first one being the acquisition of the images from different photographic angles. These images then go through a process of orientation, triangulation (calculating the position of the point or object in 3D), point-cloud generation (identifying common points among the photographs), surface reconstruction (triangulation by using the point cloud), and texture mapping, where the 3D model is finalized [84]. Nowadays these steps are done by using dedicated software [1,2].

In the neuroanatomy laboratory, photogrammetry is used to generate 3D models based on cadaveric dissections [2,18,19], which allows for their repeated use and their prolonged preservation in the virtual space and their application in residents training to acquire a better understanding of complex anatomy. The purpose of the models ranges from a more general representation of anatomy [5,7,8,19,21] to the creation of collections of specific representations of surgical approaches [13-16,20,22].

The method is gradually finding its way into clinical practice with the diagnosis of craniosynostosis and non-craniosynostosis skull deformities [23-52] and scoliosis [53-83]. This is because photogrammetry offers the possibility to estimate the shape of objects and their distance from one another. Thus, numerous researchers have started exploring its use in conditions that present themselves with superficial misalignments of body composition. The devices and software used for this purpose are constantly evolving with the advancement of technology.

Additionally, more specific cases of the use of photogrammetry in the field of neurosurgery are appearing, such as in cervical-spine surgery [85], skull-base surgery [86], and radiosurgery [87].

The purpose of this review is to summarize the uses of photogrammetry in neurosurgical practice. Furthermore, we offer insight into the systems and software required for the implementation of these techniques in everyday practice.

## Review

Methods

Literature Review

To present the uses of photogrammetry in the neurosurgical field, a narrative review was conducted on the online database PubMed. The keywords used were “photogrammetry”, “neurosurgery”, “neuroanatomy”, “craniosynostosis” and “scoliosis”. We have not limited the search results to a certain year. Nevertheless, most of the articles in the “Neuroanatomy, Surgical Anatomy” section were published in the last four years and the majority of the articles in the other sections were published after the year 2010.

Inclusion and Exclusion Criteria

The main inclusion criteria of the article correlate with the paper’s aim - to provide concise information on the uses of photogrammetry in neurosurgical education, teaching, evaluation, and diagnosis of patients. Therefore, the following inclusion criteria were used: (1) photogrammetry was the main method used in the study (model creation, patient evaluation); (2) the systems and software used for the photogrammetric technique are specified in the paper; (3) paper is written in English; and (4) the technique was applied to the field of neurosurgery. The main exclusion criteria were as follows: (1) non-photogrammetry studies; (2) studies in which photogrammetry was used for another field of medicine (3) papers not available in full text; (4) animal studies; and (5) papers that did not specify what systems and software were used for the study, therefore, the technical part of the photogrammetry process could not be presented.

**Figure 1:**
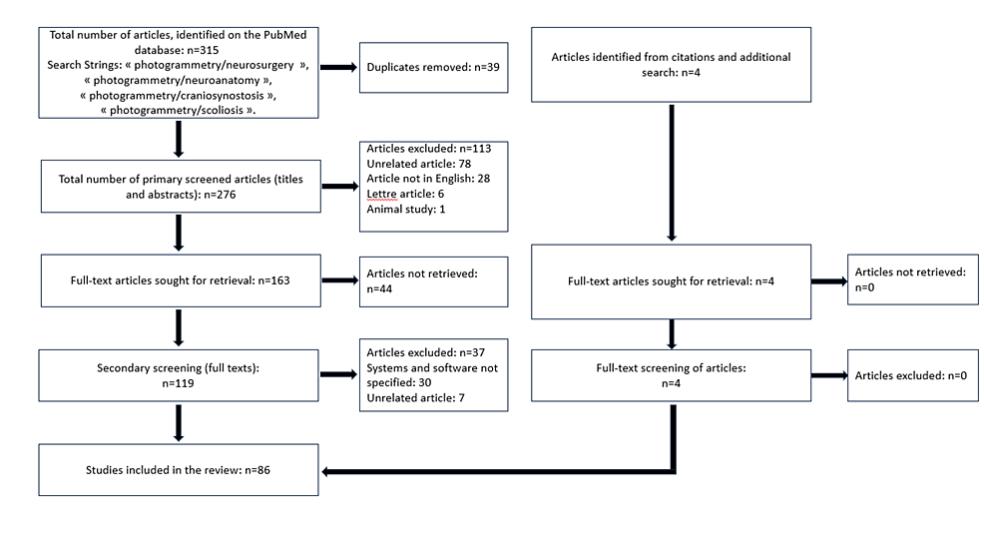
Narrative Review Flowchart Flowchart presenting the work process behind the review - the different stages of identification and screening of articles.

The papers that did not meet the criteria were excluded. The remaining studies were included in the review. We have created a flowchart that summarizes the process of screening (Figure 1).

Results

After the primary and secondary screening were conducted and the additionally identified articles included, we were left with a total of 86 studies out of the 315 initially identified. A great number of studies among the “unrelated articles” were excluded because they did not fully encompass the subject in question. This would mean that they either did not use photogrammetry, even though they were related to neurosurgery, or they used photogrammetry, however, in a completely different context.

We used the data derived from the studies to create the following table, which showcases the different contexts of use of the photogrammetry method as well as a list of the software and image acquisition systems required for its implementation (Table 1).

**Table 1:** Photogrammetry Table The table presents the systems and software identified in the four groups of studies – Neuroanatomy, Surgical Anatomy; Craniosynostosis, Skull-Shape and Development; Scoliosis and Posture; and Others. General information on the application of the method and its results are showcased. AR: augmented reality; VR: virtual reality; DSLR: digital single-lens reflex

Scope	Application	Result after image processing	Image acquisition	Image analysis software	Post-processing software	References
Neuroanatomy; Surgical Anatomy;	Education; Creation of 3D anatomical models; Clinical training; AR and VR application.	Generating a 3D anatomical model; Use of the 3D models in AR and VR. Measurement of distance, shape and motion; Exploration of a neurosurgical field.	DSLR Camera; Endoscope (Stryker, Kalamazoo, MI); VisionRT (Vision RT Ltd., London, England).	Agisoft Metashape software (Agisoft LLC, St. Petersburg, Russia); Autodesk ReCap Photo (Autodesk, San Rafael, California, USA); Reality Capture Beta 1.0 (Capturing Reality, Bratislava, Slovakia); Qlone (QloneC 2017-2020, EyeCue Vision Technologies Ltd, Yokneam, Israel); MiDaS (Intel Intelligent Systems Lab, Intel Corporation, Santa Clara, CA); Callipyan (Callipyan 3D, Copyright 2003, Robert Swirsky); Polycam (Polycam 2020-2021, Polycam Inc.).	MeshLab (Visual Computing Lab, ISTI-CNR, Pisa, Italy); Blender (Blender Foundation, Amsterdam, The Netherlands); Autodesk Meshmixer (https://meshmixer.com/); Zbrush (Pixology Inc., California, USA); Nomad (Nomad Sculpt, © Stéphane Ginier).	22 studies [1 - 22]
Craniosynostosis, Skull Shape and Skull Development	Metric analysis of 3D data; Measurement of angles, volume, shape and the surface of the object.	Diagnosis; Measuring of the cranial vault volume; Pre- and postoperative evaluation.	3DMDhead System (3dMD Inc., Atlanta, GA, USA); Canfield VECTRA-360- nine-pod system (CanfieldScientific, Parsippany, NJ, USA); Optical-Light Scanner (3D-Shape, Erlangen, Germany); STARscanner (Orthometrica Products, Inc., Orlando, FL, USA); Smartphone Camera; DSLR Camera.	VAM software (‘Visualization, Analysis, Measurement’, Fairfield, USA, Vectra); Agisoft Photoscan software (Agisoft LLC, St. Petersburg, Russia); 3dMD (3dMD Inc., Atlanta, GA, USA); Rapidform 2006 (INUS Technology, Seoul, Korea); Polycam (Polycam 2020-2021, Polycam Inc.).	Matlab (MATLAB v2012b, The Mathworks Inc., Natick, MA, USA); MeshLab (Visual Computing Lab, ISTI-CNR, Pisa, Italy); Cranioform Analytics 4.0 software(Cranioform, Alpnach, Switzerland); Blender (Blender Foundation, Amsterdam, The Netherlands); AutoCAD (Autodesk, Inc., San Francisco, CA, USA).	30 studies [23 - 52]
Scoliosis and Posture	Metric analysis of 3D data; Measurement of angles, shape and the surface of the object.	Diagnosis; Screening of patients; Posture evaluation	3D Orthoscreen (Medical University of Warsaw; 3dMDtorso (3dMD Inc., Atlanta, GA, USA); Minolta VIVID 700 Laser scanner; Formetric system (DIERS International Gmbh, Schlangenbad, Germany, Germany); Otsuka system, 1980 (Fuji Optical Co. Ltd.); DSLR Camera.	3D Orthoscreen (Medical University of Warsaw, Wojciech Glinkowski, Jakub Micho´nski);	CorelDraw13 software (Corel Corporation, Ottawa, Canada); Matlab (MATLAB v2012b, The Mathworks Inc., Natick, MA, USA).	31 studies [53 - 83]
Others	Post-operative range of motion evaluation; Intraoperative patient motion evaluation; Dural defect closure.	Function preservation evaluation; Treatment plan appropriation.	Endoscope (Karl Storz GmbH & Co. KG); VisionRT (Vision RT Ltd., London, England); DSLR Camera.	Agisoft Photoscan software (Agisoft LLC, St. Petersburg, Russia)	Autodesk Meshmixer (https://meshmixer.com/)	3 studies [85 - 87]

The articles were classified into three main groups according to their scope: Group 1 - "Neuroanatomy, Surgical Anatomy", Group 2 - "Craniosynostosis, Non-synostotic Cranial Deformities, and Skull Development", and Group 3 - "Scoliosis and Posture".

Neuroanatomy, Surgical Anatomy

In the first group, we’ve included the papers that showed the use of photogrammetry in neuroanatomy for the creation of 3D anatomical models [1-22]. All of the papers used cadavers for the creation of the models, except one, which explored the generation of intraoperative 3D models using Digital Single-Lens Reflex (DSLR) cameras [11]. For image acquisition, in the studies, the authors used DSLR cameras [3,8,9,11-16,18,19,21,22] smartphone cameras [1,2,4-8,20], and endoscope cameras [10,17]. The consensus among the studies is that anatomical models of high quality and accuracy can be created using ordinary cameras and smartphones. For the image acquisition itself, the authors recommend that the parts in shade are lightened, and the lit areas darkened so that the quality and clearance of the model are similar all around [18].

For the creation of the model from 2D pictures, different types of software exist and they usually require the use of many images showing different points of view of the object, such as Autodesk ReCap Photo (Autodesk Inc., San Francisco, CA, USA) [11,17], Reality Capture Beta (Capturing Reality, Bratislava, Slovakia) [18], and Agisoft Metashape software (Agisoft LLC, St. Petersburg, Russia) [19]. Software solutions for smartphone-based photogrammetry, such as Metascan (Abound Labs Inc., New York, NY, USA) [19] or Qlone (QloneC 2017-2020, EyeCue Vision Technologies Ltd, Yokneam, Israel) [6] have allowed moving the processing phase from the device to a cloud space which further facilitates the method. Taking the process a step further, the AI-integrated Intel ISL MiDaS v2.1 (Intel Labs, Santa Clara, CA, USA) software is capable of monocular depth estimation derived from the color intensity of every specific pixel. This creates the opportunity for 3D model creation from a single 2D photo, which according to the study could potentially close the distance between neuroimaging and photogrammetry scanning technologies [3,4,9].

For the editing of the 3D model, the latter is uploaded on a special 3D editing software, such as MeshLab (Visual Computing Lab, ISTI-CNR, Pisa, Italy) [2,3,9], Autodesk Meshmixer (https://meshmixer.com/) [18] or Blender (Blender Foundation, Amsterdam, The Netherlands) [10,11,19,20]. This step is necessary for the quality improvement of the models, which usually includes the removal of unnecessary elements [9,18,19]. Another innovative aspect of the 3D models is that they can be used in virtual reality (VR) and augmented reality (AR) environments [1,6]. With dedicated software, such as the Qlone software the model can be visualised in an AR environment. The model needs to be placed on a surface, such as a table. Furthermore, the model can be uploaded on a VR headset [1]. These advancements make the process more interactive.

The methodology for the creation of intraoperative 3D models is similar to the one used in the cadaver model creation [11]. In the study, the ReCap Pro software was used for the model creation, and Blender software for improving the model. The result is the creation of a high-quality model realistically visualizing the operative field.

Craniosynostosis, Non-synostotic Cranial Deformities, and Skull Development

The diagnosis of craniosynostosis through photogrammetry is based primarily on shape recognition. Both expensive equipment [25,32] and smartphone cameras [23] can be used for the image acquisition part of the process. Additionally, a deep-learning algorithm that uses a database of pre-uploaded head images, normal and pathological, can speed up the process [24]. Larger datasets of patients with different head shapes are necessary for the training of the algorithm’s learning capabilities, which will potentially be facilitated through the use of DSLR cameras for the image-capturing part of the process. Several papers in this section presented the use of photogrammetry in postoperative evaluation [25,26,29,33,36,44,50] which, if applied widely, can potentially reduce radiation exposure during patient follow-up, whilst maintaining objectivity. However, two studies compared the method with CT scans and reported it as a valuable alternative, not as superior [29,50].

A review article that explored the topic of photogrammetry in craniosynostosis published in the year 2022 has been identified [28]. Our review confirms the results of the paper and goes more in-depth on the software and systems used for the method. Additionally, we identified four papers published after the submission date of the aforementioned review [27,31,34,47]. Notably, among these studies, Kronig et al. propose a novel approach to diagnosing craniosynostosis through photogrammetry with the use of the Utrecht Cranial Shape Quantificator method, which was successful in diagnosing each of the five types of craniosynostosis [31]. Shaufelberger et al. employed the creation of a statistical shape model of a craniosynostosis head from a large dataset of patients which demonstrates results comparable to the CT scans [47].

The most widely used systems among the studies are the 3dMD System (3dMD Inc., Atlanta, GA, USA) [24,25,30,42], and the Canfield VECTRA-360 nine-pod system (Canfield Scientific, Parsippany, NJ, USA) [27,47,49,51], which are developed specifically for the creation of 3D models from images. The systems come with proprietary software that deals with the integration of the images into one single model. For the refinement of the model third-party software is used, such as Blender [30,42] and MeshLab [27,47]. The alternative to using such technology is the use of standard cameras and third-party software both for the creation of the model and for its refinement, which was described in one paper [23]. The method removes the need for expensive equipment and potentially incentivizes more specialists to apply it through the comfort of their mobile phones. According to studies, photogrammetry is a valuable tool for craniosynostosis evaluation and follow-up, which offers comparable results to CT without excessive radiation [37,49]. The use of photogrammetry, however, cannot yet fully replace CT scans as it has demonstrated limited diagnosis capabilities in milder cases of the condition [30].

When evaluating non-synostotic cranial deformities, which are becoming more prevalent with the infants’ supine position (positional plagiocephaly), commonly chosen to prevent sudden infant death syndrome), photogrammetry demonstrated good results for the diagnosis, treatment planning, and follow-up of the patients [45,46]. Any deviations in the process of skull development can lead to esthetical and psychological problems later in life. By making measurements through photogrammetry at two different points in time the development of the skull can be assessed and any deviation from the normal growth can be detected early on [27,39,40].

Scoliosis and Posture

Photogrammetry has been used for the detection of scoliosis for a couple of decades now. The first instances involve the use of the “Moiré topography”, a method utilizing the projection of a series of light stripes over the patient’s back [64,65,74,82,83]. Later the methods evolved through the use of “Rasterstereography”, which involves the use of a special system - the Formetric system (DIERS International Gmbh, Schlangenbad, Germany), to capture a series of pictures of the back of the patient from different angles, which are later analyzed by dedicated software [55,63,67,78]. Nowadays technology has progressed to allow the use of DSLR cameras for the purpose of image acquisition along with dedicated software to analyze the data [57,58,67,69].

When comparing the photogrammetric method with the radiographic method, it reflected accurately the progression of lateral vertebral deviation and vertebral rotation. [78,80]. The method cannot replace radiography, however, it can potentially be applied in the follow-up of patients to dilute the number of radiographs to once every 12 to 18 months, provided the photogrammetric evaluation does not show rapid worsening of scoliosis. The method has been applied as a non-ionizing means of school screening of children for scoliosis [58,61]. The main limitations of photogrammetry in that aspect arise with individuals of high Body Mass Index, due to the greater challenge in localizing the spinous processes. Furthermore, the surgical removal of the spinous processes renders the method unusable, since the anatomical structure used to make the measurement is absent in these patients [58].

Photogrammetry is also a reliable method to evaluate different parameters of the human posture, such as the trunk length and inclination, as well as the kyphotic and lordotic angles [68,71,72]. The use of machine learning algorithms could hypothetically facilitate even further the assessment and bring it to the level of comfort of a mobile application [69]. Through the use of an AI-powered application, with the automatic identification and analysis of "Anatomical and Segment Points", points correlating with anatomical sites, such as the trochanters, anterior superior iliac spine, and the acromion, the detection of postural abnormalities, joint/muscle impediments, range of motion, and the monitoring of patient rehabilitation could be assisted. Nevertheless, further studies are needed for the smoother implementation of such an application in clinical practice.

Other Applications

We have identified three distinct papers showcasing the use of photogrammetry for range of motion assessment in cervical spine surgery, skull-base surgery, and radiosurgery.

In cervical spine surgery, laminoplasty is a procedure used to treat multilevel spine stenosis and is associated with a change in the range of motion of the spine postoperatively. Photogrammetry can be used as an inexpensive and radiation-free method to measure the exact extent of this change in all planes [85]. The study used pre- and postoperative images taken by DSLR camera. The chin-brow vertical angle was measured during flexion and extension for the sagittal plane and the nose-turn angle was used for the axial plane. In skull-base surgery, one of the major challenges is the closure of dural defects. This defect is usually fixed either through mobilizing the pericranium (autograft) or by using artificial implants. The size of the material is usually subjectively assessed by the operator. Through the use of photogrammetry, the size of the implant can be accurately calculated using contour and vertex color to determine the size of the defect [86]. In radiosurgery, photogrammetry has been used to assess the effect on the outcome of the movement of the patient’s head during the procedure [87]. The VisionRT (VisionRT Ltd., London, UK) system was used for the purpose. The study concluded that treatment plans that target multiple metastases by using the same point of radiation are more sensitive to patient movement.

Discussion

In this paper, we have evaluated the application of a relatively novel (for neurosurgery) algorithm for surface scanning, photogrammetry, and its various applications to neurosurgical practice, including mainly neurosurgical anatomy but also various potential clinical applications, such as cranial deformity and scoliosis evaluation, where the method can potentially reduce radiation exposure.

Neurosurgical Anatomy Training

Studying the complex anatomy of the nervous system is a long and difficult process. Answering the need to diversify the methods for this purpose, photogrammetry has been applied for the creation of accurate 3D models of different anatomical components.

The studies in this review show that both general neuroanatomy models and specific neurosurgical models and simulations of approaches can be created with the photogrammetry method [1-22].

The development of 3D models in neuroanatomical teaching can potentially revolutionize the field since it offers a solution to many of the problems that standard teaching methods are facing. The methods offer the possibility to manipulate the model by zooming in and out of regions of interest to study the specific, often annotated structures [1,2,20]. Through this, the examiner can acquire knowledge about the actual proportions and depth of a specific model, the distance between different structures, and their respective positions in relation to other structures. This gives the advantage of the 3D models when compared with the conventional 2D images used for neuroanatomy teaching. Both medical students and neurosurgery residents can use the models to learn the complex anatomy of the nervous system and spatial orientation during cranial surgery [17].

The introduction of the models in VR and AR environments makes the process more interactive. Through AR, the user can project the models on any surface, enlarge them, and look around every detail of the model [1,6]. With a VR headset, the user can create a virtual neuroanatomy lab [1,4,17] and examine the models with greater ease. The VR device offers the possibility to grab the model and manipulate it in every plane, thus facilitating the process of examination.

Furthermore, many of the logistical difficulties associated with acquiring cadavers for neuroanatomy training, such as the cost and the ethical concerns are in great part dealt with through the use of the 3D models. The lack of cadavers in neuroanatomy laboratories makes the easy distribution of the models an even more valuable trait. With time the deterioration of the models and the difficulties associated with their conservation is another burden for current anatomy departments. When a 3D model is uploaded on a platform it can remain there for an indefinite period of time, thus the conservation of a valuable anatomical model and a potentially rare anatomical variant is facilitated through the use of the photogrammetric technique.

**Figure 2:**
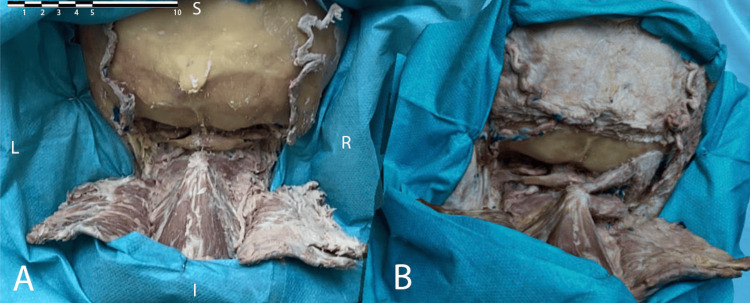
Screenshot of a direct comparison of real anatomical dissection of posterior neck region (A) and augmented reality model (B) placed next to each other to demonstrate the level of photorealism of the 3D model. 3D - Three-dimensional; L - left; R - Right; S - Superior; I - Inferior The scale bar is in centimeters (upper left corner). Published with the permission of the "3D Atlas of Neurological Surgery" platform [88].

**Figure 3:**
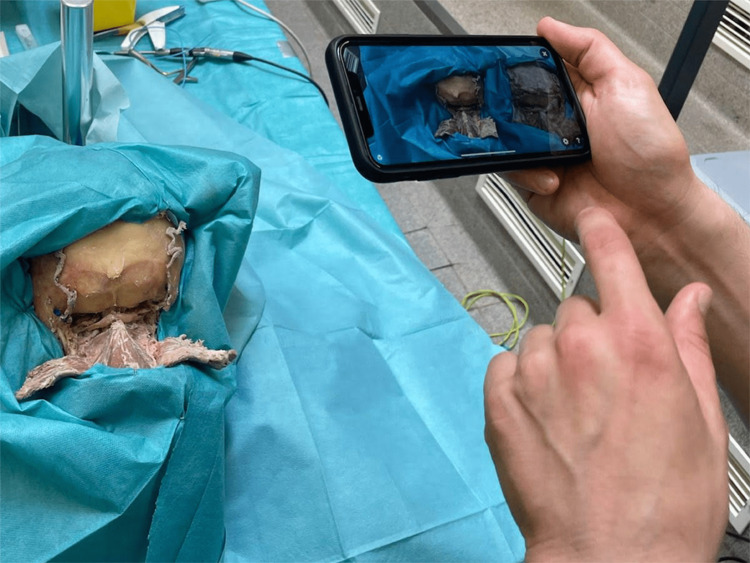
Photo demonstrating the real view of the observed augmented reality settings in the anatomical laboratory. 3D - Three-dimensional Published with the permission of the "3D Atlas of Neurological Surgery" platform [88].

**Figure 4:**
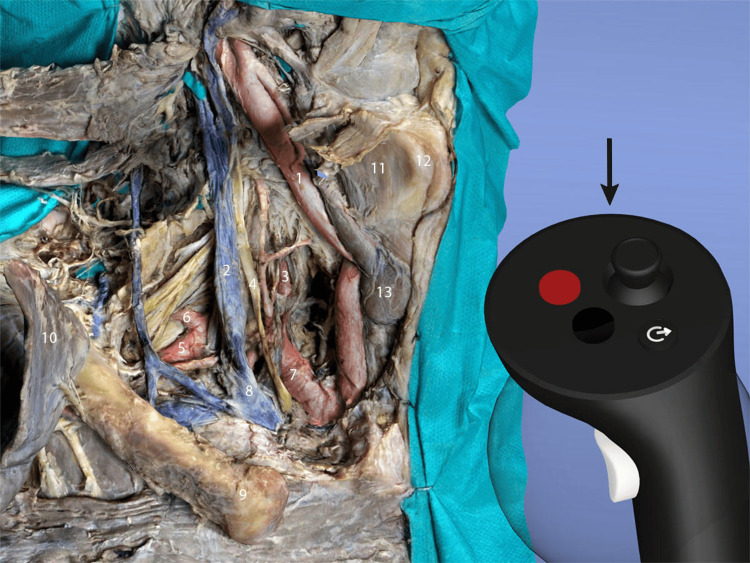
Immersive virtual reality (VR) test of a photorealistic model of anterior neck dissection loaded in the platform Gravity Sketch (Gravity Sketch, 2014, https://www.gravitysketch.com/). 3D - Three-dimensional Gravity Sketch is an innovative VR 3D modeling application. The model can be rotated, zoomed, and observed from different angles in a completely immersive environment with the help of the device's controllers (black arrow). The vessels and the nerves of the 3D model are additionally colored in Blender software (Blender Foundation, Amsterdam, The Netherlands, https://www.blender.org). 1 - Common Carotid Artery; 2 - Internal Jugular Vein; 3 - Vertebral Artery; 4 - Vagus Nerve; 5 - Subclavian Artery; 6 - Transverse ; Cervical Artery; 7 - Brachiocephalic Trunk; 8 - Brachiocephalic Vein; 9 - Clavicle; 10 - Pectoralis Major Muscle; 11 - Thyrohyoid Muscle; 12 - Thyroid Cartilage; 13 - Thyroid Gland Published with the permission of the "3D Atlas of Neurological Surgery" platform [88].

The studies show the increasing use of photogrammetry in neurosurgical anatomy as it provides authenticity and photorealism to 3D models [1,2]. The resulting 3D photorealistic models can be used for anatomical and neurosurgical training in augmented (Figures 2, 3) and virtual reality (Figure 4). When a 3D model is uploaded on a platform it can remain there for an indefinite period of time, thus the conservation of a valuable anatomical model and a potentially rare anatomical variant is facilitated through the use of the photogrammetric technique.

Through the application of the photogrammetric method, a collection of immersive surgical anatomy of different neurosurgical approaches has been already published in the literature [10,13-16,18,21,22]. There are very detailed papers for cadaver-based models for complex approaches such as Far-Lateral [13], Frontotemporal-Orbitozygomatic [14], Pterional [15], Retrosigmoid [16], and the Smith-Robinson approach to the anterior spine [22]. Despite that, cadaver training remains essential in neurosurgery resident’s education and the new technologies can be regarded as a supplement to traditional methods of teaching and training [89]. Therefore photogrammetry-based models and a new training curriculum in VR can be a good supplement to traditional training methods [4]. In their studies, Aydin et al. and Gonzales-Romo et al. have created virtual neuroanatomy laboratories where the models are uploaded and examined [1,4]. According to the authors, before the application of photogrammetry, the only available option for studying neuroanatomy in AR and VR was through models produced by images and drawings in computer programs, which are limited by the artist’s capability and anatomical knowledge. Nowadays, with the creation of 3D models from cadaver dissections, the method is becoming more accurate since it no longer depends on a non-specialized point of view of the captured field. The reported limitations are loss of image quality and long durations of the creation process of the models.

Combining photogrammetry anatomical scans with CT or MRI-segmented 3D data is another innovation that can potentially improve neurosurgical training. Hanalioglu et al. used an integrated method between 3D reconstructions of MRI and CT scans, neuronavigation, and photogrammetry to create quantitatively and qualitatively accurate 3D models of the pterional craniotomy [9]. The model allows for a layer-by-layer simulation of the technique. However, one of the drawbacks of the method is that cadaver neuroimaging doesn’t allow for visualization of the vessels, thus they had to be rendered with the help of the MeshLab Software. Another study by Roh et al. used photogrammetry brain scans combined with a CT-based 3D model of a skull to simulate neurosurgical approaches in a VR environment [17]. Despite the obvious advantages of the method, there was no tactile feedback or the possibility to simulate surgical instruments. All these studies concerning neurosurgical anatomy may revolutionize the way anatomy is taught in the next few years and potentially improve the quality of education in places where cadaver training is limited.

Clinical Application

Two distinct pathologies concerning neurosurgery, characterized by deformation of the surface details of body parts are craniosynostosis [23-52] and scoliosis [53-83] are currently studied by photogrammetry. There is growing data that photogrammetry can be used to diagnose, follow up, and screen these conditions. This is possible since both craniosynostosis and scoliosis are conditions, associated with surface deformation of the shape of the head and spine respectively. Furthermore, the current standards for diagnosing and following these conditions are largely associated with radiation exposure to the patients (predominantly children). When applied with the right technique and software, photogrammetry offers the possibility of shape evaluation without the excessive radiation burden [38,57,58].

Generally, when managing craniosynostosis, anthropometric measurements are used for pre- and postoperative evaluation. However, they are time-consuming and rely on the physician’s expertise which is subjective and largely depends on experience. A more objective method is the use of CT to confirm the diagnosis. CT however is associated with radiation exposure.

Most of the studies described the use of expensive systems for the acquisition of images [24,25,47,49]. One study, however, described the possibility of replacing expensive equipment with ordinary smartphone technology [23]. The authors explain that using a DSLR camera has the limitations of appropriate lighting combined with a static patient, which is difficult in the pediatric patient population unless anesthesia is applied. Using a standard smartphone camera for the acquisition of slow-motion videos was the method of choice, which provided a decent number of suitable images of different angles, necessary for the creation of the 3D model. A cap with stickers working as targets was also put on the infant’s head, on one hand, to prevent the hair from getting in the way of the model creation and on the other because the targets facilitate the model creation. Overall, the image acquisition required three to five minutes without the use of any special equipment, considering the fact that the patient was held in place by an adult.

No study demonstrated a comparison of the different camera systems used for the acquisition of the images, thus no consensus has so far been reached on which is the most suitable one. Future research should be directed toward a comparison of the different imaging systems and software for the evaluations of cranial deformities.

The diagnosis and evaluation of scoliosis and its severity are crucial steps in the treatment strategy of the condition. The method of choice is usually radiography, through which the Cobb angle is measured. It gives insight into the progression of the condition and helps in planning the intervention if needed. The drawback of the method is the radiation exposure of the patients. The use of photogrammetry in scoliosis diagnosis relies on the measurement of the angles between various points of the spine, such as the angles between the vertebral bodies and the angles between the vertebral body and the horizontal plane [70]. Differences in the height of the shoulders and the prominence of the waist are also observed.

Another method that precludes radiation exposure is the use of a scoliometer that can accurately measure the angle of trunk rotation in scoliosis patients. Photogrammetry has been compared with the scoliometer for its ability to measure the angle of trunk rotation when applied correctly [70]. The authors used a DSLR camera and the so-calledDIPA (Digital Image-based Postural Assessment) software (The Mathworks Inc., Natick, MA, USA), which is a dedicated software for the analysis of posture on images. After acquiring the data and comparing it with the scoliometer measurements, the two methods showed a high correlation with an error of 3° and a significance level of P ≤ .05. Thus, the method can be adequately used for the diagnosis of mild to severe scoliosis and with the added benefit of preserving a photograph of the patient with the applied measurements for future reference if needed. However, according to the data and the protocols that are published up until now, the method cannot replace the radiography, it can only be used as a supplement to it. Further improvement is needed before the complete replacement of radiography by surface scanning is deemed adequate.

With the advancement of the method, numerous other uses of photogrammetry are appearing, such as in cervical-spine surgery after laminoplasty [85], in skull-base surgery for repairment of the dural defect [86], as well as in radiosurgery intraoperatively [87].

All these applications of the technology in the neurosurgical field show its potential and the evolution of the photogrammetric technique used for these purposes has been steady over the past few years. Starting from expensive and complex devices [30,81] it has now arrived at the point where a regular DSLR camera [3,18,19,22] or even a smartphone [6,20,23,69,90] with cloud computing and dedicated software can be used to accomplish the tasks in minutes.

Limitations

The major limitation of our study is the use of a singular database - PubMed. Although PubMed has been widely regarded as one of the most comprehensive biomedical literature sources, a systematic review featuring a larger number of databases and registers could potentially offer a wider perspective of the subject.

## Conclusions

The results of this review demonstrate that the application of photogrammetry in neurosurgery is promising. The method is used in the educational setting for the creation of normal and surgical anatomy 3D models. The models show high accuracy and correlation with the anatomy of interest, are also relatively simple to create, and can be uploaded to VR and AR environments, thus offering a new mode of education for residents and medical students.

Furthermore, in the clinical setting, photogrammetry offers a non-invasive and non-ionizing method for the diagnosis and evaluation of conditions associated with head and spine deformation. Through the acquisition of images and their analysis with dedicated software, a diagnosis and assessment value in most aspects similar to that of radiographs and CT scans can be achieved, without the excessive radiation burden. Nevertheless, the method requires further improvements and research, particularly associated with its use in clinical applications.

## References

[1] Aydin SO, Barut O, Yilmaz MO (2023). Use of 3-dimensional modeling and augmented/virtual reality applications in microsurgical neuroanatomy training. Oper Neurosurg (Hagerstown).

[2] de Oliveira AS, Leonel LC, LaHood ER (2023). Foundations and guidelines for high-quality three-dimensional models using photogrammetry: a technical note on the future of neuroanatomy education. Anat Sci Educ.

[3] Gonzalez-Romo NI, Hanalioglu S, Mignucci-Jiménez G, Abramov I, Xu Y, Preul MC (2023). Anatomic depth estimation and 3-dimensional reconstruction of microsurgical anatomy using monoscopic high-definition photogrammetry and machine learning. Oper Neurosurg (Hagerstown).

[4] Gonzalez-Romo NI, Mignucci-Jiménez G, Hanalioglu S (2023). Virtual neurosurgery anatomy laboratory: a collaborative and remote education experience in the metaverse. Surg Neurol Int.

[5] Gurses ME, Gungor A, Gökalp E (2022). Three-dimensional modeling and augmented and virtual reality simulations of the white matter anatomy of the cerebrum. Oper Neurosurg (Hagerstown).

[6] Gurses ME, Gungor A, Hanalioglu S, Yaltirik CK, Postuk HC, Berker M, Türe U (2021). Qlone®: a simple method to create 360-degree photogrammetry-based 3-dimensional model of cadaveric specimens. Oper Neurosurg (Hagerstown).

[7] Gurses ME, Gungor A, Rahmanov S (2022). Three-dimensional modeling and augmented reality and virtual reality simulation of fiber dissection of the cerebellum and brainstem. Oper Neurosurg (Hagerstown).

[8] Gurses ME, Hanalioglu S, Mignucci-Jiménez G (2023). Three-dimensional modeling and extended reality simulations of the cross-sectional anatomy of the cerebrum, cerebellum, and brainstem. Oper Neurosurg (Hagerstown).

[9] Hanalioglu S, Romo NG, Mignucci-Jiménez G (2022). Development and validation of a novel methodological pipeline to integrate neuroimaging and photogrammetry for immersive 3D cadaveric neurosurgical simulation. Front Surg.

[10] Kournoutas I, Vigo V, Chae R (2019). Acquisition of volumetric models of skull base anatomy using endoscopic endonasal approaches: 3D scanning of deep corridors via photogrammetry. World Neurosurg.

[11] Nicolosi F, Spena G (2020). Three-dimensional virtual intraoperative reconstruction: a novel method to explore a virtual neurosurgical field. World Neurosurg.

[12] Párraga RG, Possatti LL, Alves RV, Ribas GC, Türe U, de Oliveira E (2016). Microsurgical anatomy and internal architecture of the brainstem in 3D images: surgical considerations. J Neurosurg.

[13] Payman A, Rios Zermeno J, Hirpara A, El-Sayed IH, Abla A, Rodriguez Rubio R (2022). Immersive surgical anatomy of the far-lateral approach. Cureus.

[14] Patra A, Singla RK, Mathur M, Chaudhary P, Singal A, Asghar A, Malhotra V (2021). Morphological and morphometric analysis of the orbital aperture and their correlation with age and gender: a retrospective digital radiographic study. Cureus.

[15] Rodriguez Rubio R, Chae R, Vigo V, Abla AA, McDermott M (2019). Immersive surgical anatomy of the pterional approach. Cureus.

[16] Rodriguez Rubio R, Xie W, Vigo V, Lee A, Tomasi OS, El-Sayed IH, Abla A (2021). Immersive surgical anatomy of the retrosigmoid approach. Cureus.

[17] Roh TH, Oh JW, Jang CK, Choi S, Kim EH, Hong CK, Kim SH (2021). Virtual dissection of the real brain: integration of photographic 3D models into virtual reality and its effect on neurosurgical resident education. Neurosurg Focus.

[18] Rubio RR, Shehata J, Kournoutas I (2019). Construction of neuroanatomical volumetric models using 3-dimensional scanning techniques: technical note and applications. World Neurosurg.

[19] Spiriev T, Mitev A, Stoykov V, Dimitrov N, Maslarski I, Nakov V (2022). Three-dimensional immersive photorealistic layered dissection of superficial and deep back muscles: anatomical study. Cureus.

[20] Spiriev T, Nakov V, Cornelius JF (2023). Photorealistic 3-dimensional models of the anatomy and neurosurgical approaches to the V2, V3, and V4 segments of the vertebral artery. Oper Neurosurg (Hagerstown).

[21] Vigo V, Hirpara A, Yassin M (2020). Immersive surgical anatomy of the craniocervical junction. Cureus.

[22] Vigo V, Pastor-Escartín F, Doniz-Gonzalez A (2020). The smith-robinson approach to the subaxial cervical spine: a stepwise microsurgical technique using volumetric models from anatomic dissections. Oper Neurosurg (Hagerstown).

[23] Barbero-García I, Lerma JL, Marqués-Mateu Á, Miranda P (2017). Low-cost smartphone-based photogrammetry for the analysis of cranial deformation in infants. World Neurosurg.

[24] de Jong G, Bijlsma E, Meulstee J (2020). Combining deep learning with 3D stereophotogrammetry for craniosynostosis diagnosis. Sci Rep.

[25] de Jong G, Tolhuisen M, Meulstee J (2017). Radiation-free 3D head shape and volume evaluation after endoscopically assisted strip craniectomy followed by helmet therapy for trigonocephaly. J Craniomaxillofac Surg.

[26] Delye HH, Arts S, Borstlap WA (2016). Endoscopically assisted craniosynostosis surgery (EACS): the craniofacial team Nijmegen experience. J Craniomaxillofac Surg.

[27] Dieks JK, Jünemann L, Hensel KO (2022). Stereophotogrammetry can feasibly assess 'physiological' longitudinal three-dimensional head development of very preterm infants from birth to term. Sci Rep.

[28] Duncan C, Pears NE, Dai H, Smith WA, O'Higgins P (2022). Applications of 3D photography in craniofacial surgery. J Pediatr Neurosci.

[29] Freudlsperger C, Steinmacher S, Bächli H, Somlo E, Hoffmann J, Engel M (2015). Metopic synostosis: measuring intracranial volume change following fronto-orbital advancement using three-dimensional photogrammetry. J Craniomaxillofac Surg.

[30] Ho OA, Saber N, Stephens D, Clausen A, Drake J, Forrest C, Phillips J (2017). Comparing the use of 3D photogrammetry and computed tomography in assessing the severity of single-suture nonsyndromic craniosynostosis. Plast Surg (Oakv).

[31] Kronig SA, Kronig OD, Vrooman HA, Van Adrichem LN (2023). Classification of skull shape deformities related to craniosynostosis on 3D photogrammetry. J Craniofac Surg.

[32] Kunz F, Schweitzer T, Große S (2019). Head orthosis therapy in positional plagiocephaly: longitudinal 3D-investigation of long-term outcomes, compared with untreated infants and with a control group. Eur J Orthod.

[33] Linden OE, Baratta VM, Gonzalez JA, Byrne ME, Klinge PM, Sullivan SR, Taylor HO (2019). Surgical correction of metopic craniosynostosis: a 3-D photogrammetric analysis of cranial vault outcomes. Cleft Palate Craniofac J.

[34] Srivastava M, Asghar A, Srivastava NN, Gupta N, Jain A, Verma J (2018). An anatomic morphological study of occipital spurs in human skulls. J Craniofac Surg.

[35] Linz C, Schweitzer T, Brenner LC, Kunz F, Meyer-Marcotty P, Wermke K (2018). Does shape affect function? Articulatory skills in babbling of infants with deformational plagiocephaly. Childs Nerv Syst.

[36] Martini M, Schulz M, Röhrig A, Nadal J, Messing-Jünger M (2015). A 3D morphometric follow-up analysis after frontoorbital advancement in non-syndromic craniosynostosis. J Craniomaxillofac Surg.

[37] Mertens C, Wessel E, Berger M (2017). The value of three-dimensional photogrammetry in isolated sagittal synostosis: Impact of age and surgical technique on intracranial volume and cephalic index─a retrospective cohort study. J Craniomaxillofac Surg.

[38] Meulstee JW, Verhamme LM, Borstlap WA (2017). A new method for three-dimensional evaluation of the cranial shape and the automatic identification of craniosynostosis using 3D stereophotogrammetry. Int J Oral Maxillofac Surg.

[39] Meyer-Marcotty P, Böhm H, Linz C, Kochel J, Stellzig-Eisenhauer A, Schweitzer T (2014). Three-dimensional analysis of cranial growth from 6 to 12 months of age. Eur J Orthod.

[40] Meyer-Marcotty P, Böhm H, Linz C (2014). Spectrum of positional deformities - is there a real difference between plagiocephaly and brachycephaly?. J Craniomaxillofac Surg.

[41] Meyer-Marcotty P, Kunz F, Schweitzer T, Wachter B, Böhm H, Waßmuth N, Linz C (2018). Cranial growth in infants─a longitudinal three-dimensional analysis of the first months of life. J Craniomaxillofac Surg.

[42] Peterson EC, Patel KB, Skolnick GB, Pfeifauf KD, Davidson KN, Smyth MD, Naidoo SD (2018). Assessing calvarial vault constriction associated with helmet therapy in deformational plagiocephaly. J Neurosurg Pediatr.

[43] Sakar M, Haidar H, Sönmez Ö, Erdoğan O, Saçak B, Bayri Y, Dağçınar A (2022). A new method for quantification of frontal retrusion and complex skull shape in metopic craniosynostosis: a pilot study of a new outcome measure for endoscopic strip craniectomy. J Neurosurg Pediatr.

[44] Salokorpi N, Vuollo V, Sinikumpu JJ (2017). Increases in cranial volume with posterior cranial vault distraction in 31 consecutive cases. Neurosurgery.

[45] Schaaf H, Pons-Kuehnemann J, Malik CY, Streckbein P, Preuss M, Howaldt HP, Wilbrand JF (2010). Accuracy of three-dimensional photogrammetric images in non-synostotic cranial deformities. Neuropediatrics.

[46] Schaaf H, Malik CY, Streckbein P, Pons-Kuehnemann J, Howaldt HP, Wilbrand JF (2010). Three-dimensional photographic analysis of outcome after helmet treatment of a nonsynostotic cranial deformity. J Craniofac Surg.

[47] Schaufelberger M, Kühle R, Wachter A (2022). A radiation-free classification pipeline for craniosynostosis using statistical shape modeling. Diagnostics (Basel).

[48] Schweitzer T, Böhm H, Linz C (2013). Three-dimensional analysis of positional plagiocephaly before and after molding helmet therapy in comparison to normal head growth. Childs Nerv Syst.

[49] Seeberger R, Hoffmann J, Freudlsperger C, Berger M, Bodem J, Horn D, Engel M (2016). Intracranial volume (ICV) in isolated sagittal craniosynostosis measured by 3D photocephalometry: a new perspective on a controversial issue. J Craniomaxillofac Surg.

[50] van Veelen MC, Jippes M, Carolina JA, de Rooi J, Dirven CM, van Adrichem LN, Mathijssen IM (2016). Volume measurements on three-dimensional photogrammetry after extended strip versus total cranial remodeling for sagittal synostosis: a comparative cohort study. J Craniomaxillofac Surg.

[51] Wilbrand JF, Howaldt HP, Reinges M, Christophis P (2016). Surgical correction of lambdoid synostosis - new technique and first results. J Craniomaxillofac Surg.

[52] Wilbrand JF, Szczukowski A, Blecher JC, Pons-Kuehnemann J, Christophis P, Howaldt HP, Schaaf H (2012). Objectification of cranial vault correction for craniosynostosis by three-dimensional photography. J Craniomaxillofac Surg.

[53] Adler NS, Csongradi J, Bleck EE (1984). School screening for scoliosis—one experience in California using clinical examination and Moiré photography. West J Med.

[54] Andonian AT (1984). Detection of stimulated back muscle contractions by moiré topography. J Biomech.

[55] Applebaum A, Ference R, Cho W (2020). Evaluating the role of surface topography in the surveillance of scoliosis. Spine Deform.

[56] Aroeira RM, de Las Casas EB, Pertence AE, Greco M, Tavares JM (2016). Non-invasive methods of computer vision in the posture evaluation of adolescent idiopathic scoliosis. J Bodyw Mov Ther.

[57] Aroeira RM, Leal JS, de Melo Pertence AE (2011). New method of scoliosis assessment: preliminary results using computerized photogrammetry. Spine (Phila Pa 1976).

[58] Aroeira RM, Leal JS, Pertence AE, Casas EB, Greco M (2019). Non-ionizing method of screening adolescent idiopathic scoliosis in schoolchildren. Cien Saude Colet.

[59] Batouche M, Benlamri R, Kholladi MK (1996). A computer vision system for diagnosing scoliosis using moiré images. Comput Biol Med.

[60] Berg DC, Hill DL, Raso VJ, Lou E, Church T, Moreau MJ, Mahood JK (2002). Using three-dimensional difference maps to assess changes in scoliotic deformities. Med Biol Eng Comput.

[61] Drzał-Grabiec J, Snela S, Podgórska-Bednarz J, Rykała J, Banaś A (2014). Examination of the compatibility of the photogrammetric method with the phenomenon of mora projection in the evaluation of scoliosis. Biomed Res Int.

[62] Gabor LR, Chamberlin AP, Levy E, Perry MB, Cintas H, Paul SM (2011). Digital stereophotogrammetry as a new technique to quantify truncal deformity: a pilot study in persons with osteogenesis imperfecta. Am J Phys Med Rehabil.

[63] Girdler S, Cho B, Mikhail CM, Cheung ZB, Maza N, Kang-Wook Cho S (2020). Emerging techniques in diagnostic imaging for idiopathic scoliosis in children and adolescents: a review of the literature. World Neurosurg.

[64] Kim HS, Ishikawa S, Ohtsuka Y, Shimizu H, Shinomiya T, Viergever MA (2001). Automatic scoliosis detection based on local centroids evaluation on moiré topographic images of human backs. IEEE Trans Med Imaging.

[65] Labecka MK, Plandowska M (2021). Moiré topography as a screening and diagnostic tool-a systematic review. PLoS One.

[66] Leal JS, Aroeira RM, Gressler V, Greco M, Pertence AE, Lamounier JA (2019). Accuracy of photogrammetry for detecting adolescent idiopathic scoliosis progression. Spine J.

[67] Mangone M, Raimondi P, Paoloni M (2013). Vertebral rotation in adolescent idiopathic scoliosis calculated by radiograph and back surface analysis-based methods: correlation between the Raimondi method and rasterstereography. Eur Spine J.

[68] Mohokum M, Mendoza S, Udo W, Sitter H, Paletta JR, Skwara A (2010). Reproducibility of rasterstereography for kyphotic and lordotic angles, trunk length, and trunk inclination: a reliability study. Spine (Phila Pa 1976).

[69] Moreira R, Teles A, Fialho R (2020). Can human posture and range of motion be measured automatically by smart mobile applications?. Med Hypotheses.

[70] Navarro IJ, Candotti CT, do Amaral MA, Dutra VH, Gelain GM, Loss JF (2020). Validation of the measurement of the angle of trunk rotation in photogrammetry. J Manipulative Physiol Ther.

[71] Navarro IJ, Candotti CT, Furlanetto TS, Dutra VH, do Amaral MA, Loss JF (2019). Validation of a mathematical procedure for the Cobb angle assessment based on photogrammetry. J Chiropr Med.

[72] Paul SM, Chamberlin AP, Hatt C, Nayak AV, Danoff JV (2009). Reliability, validity, and precision of an active stereophotogrammetry system for three-dimensional evaluation of the human torso. Med Eng Phys.

[73] Penha PJ, Penha NL, De Carvalho BK, Andrade RM, Schmitt AC, João SM (2017). Posture alignment of adolescent idiopathic scoliosis: photogrammetry in scoliosis school screening. J Manipulative Physiol Ther.

[74] Porto F, Gurgel JL, Russomano T, Farinatti Pde T (2010). Moiré topography: characteristics and clinical application. Gait Posture.

[75] Saad KR, Colombo AS, João SM (2009). Reliability and validity of the photogrammetry for scoliosis evaluation: a cross-sectional prospective study. J Manipulative Physiol Ther.

[76] Saad KR, Colombo AS, Ribeiro AP, João SM (2012). Reliability of photogrammetry in the evaluation of the postural aspects of individuals with structural scoliosis. J Bodyw Mov Ther.

[77] Schroeder J, Reer R, Braumann KM (2015). Video raster stereography back shape reconstruction: a reliability study for sagittal, frontal, and transversal plane parameters. Eur Spine J.

[78] Schulte TL, Hierholzer E, Boerke A, Lerner T, Liljenqvist U, Bullmann V, Hackenberg L (2008). Raster stereography versus radiography in the long-term follow-up of idiopathic scoliosis. J Spinal Disord Tech.

[79] Stolinski L, Kozinoga M, Czaprowski D, Tyrakowski M, Cerny P, Suzuki N, Kotwicki T (2017). Two-dimensional digital photography for child body posture evaluation: standardized technique, reliable parameters and normative data for age 7-10 years. Scoliosis Spinal Disord.

[80] Terheyden JH, Wetterkamp M, Gosheger G, Lange T, Schulze Bövingloh A, Schulte TL (2018). Rasterstereography versus radiography for assessing shoulder balance in idiopathic scoliosis: a validation study relative to patients' self-image. J Back Musculoskelet Rehabil.

[81] van Wijk MC (1980). Moiré contourgraph--an accuracy analysis. J Biomech.

[82] Willner S (1979). Moiré topography--a method for school screening of scoliosis. Arch Orthop Trauma Surg (1978).

[83] Willner S (1979). Moiré topography for the diagnosis and documentation of scoliosis. Acta Orthop Scand.

[84] Ozsoy U, Sekerci R, Ogut E (2015). Effect of sitting, standing, and supine body positions on facial soft tissue: detailed 3D analysis. Int J Oral Maxillofac Surg.

[85] Janjua MB, Zhou PL, Vasquez-Montes D, Moskovich R (2020). Photogrammetric analysis: an objective measure to assess the craniocervical range of motion after cervical laminoplasty surgeries. J Clin Neurosci.

[86] Shin J, Forbes J, Lehner K, Tomasiewicz H, Schwartz TH, Phillips CD (2019). Skull base 3D modeling of rigid buttress for gasket-seal closure using operative endoscopic imaging: cadaveric feasibility. J Neurol Surg B Skull Base.

[87] Yock AD, Pawlicki T, Kim GY (2016). Prospective treatment plan-specific action limits for real-time intrafractional monitoring in surface image guided radiosurgery. Med Phys.

[88] (2023). 3D atlas of neurological surgery. https://3datlasofneurologicalsurgery.org/.

[89] Stengel FC, Gandia-Gonzalez ML, Aldea CC (2022). Transformation of neurosurgical training from "see one, do one, teach one" to AR/VR & simulation - a survey by the EANS Young Neurosurgeons. Brain Spine.

[90] Krogager ME, Fugleholm K, Mathiesen TI, Spiriev T (2023). Simplified easy-accessible smartphone-based photogrammetry for 3-dimensional anatomy presentation exemplified with a photorealistic cadaver-based model of the intracranial and extracranial course of the facial nerve. Oper Neurosurg (Hagerstown).

